# Neuroprotective effects of semaglutide and metformin against rotenone-induced neurobehavioral changes in male diabetic rats

**DOI:** 10.1007/s00210-025-03920-7

**Published:** 2025-03-15

**Authors:** Esraa A. Salem, Saad Misfer Alqahtani, Ehab A. M. El-Shoura, Sameh S. Zaghlool, Lobna A. Abdelzaher, Sally A. M. Mohamed, Ibrahim S. Alalhareth, Alzahraa A. M. Sheref

**Affiliations:** 1 Department of Medical Physiology, Faculty of Medicine, Menoufia University, Shebeen ElKom, 32511 Egypt; 2 Department of Pathology, College of Medicine, The University Hospital, Najran University, Najran, Saudi Arabia; 3 Department of Clinical Pharmacy, Faculty of Pharmacy, Al-Azhar University, Assiut Branch, Assiut, Egypt; 4 Department of Pharmacology and Toxicology, Faculty of Pharmacy, Modern University of Technology and Information (MTI), Mokattam Cairo, 11571 Egypt; 5 Department of Pharmacology, Faculty of Medicine, Assiut University, Assiut, Egypt; 6 Department of Histology and Cytology, Faculty of Veterinary Medicine, Zagazig University, Zagazig, Egypt; 7 College of Pharmacy, The University Hospital, Najran University, Najran, Saudi Arabia

**Keywords:** Diabetes, Metformin, Semaglutide, Parkinson, CX3Cl1, Nrf2, GFAP

## Abstract

Pre-existing diabetes raises the likelihood of Parkinson’s disease (PD), according to epidemiological and animal research. Our study aimed to investigating the likely neuroprotective effect of metformin (Met) and/or semaglutide (Sem) in model of PD in male diabetic rats and the possible underlying mechanism. Type 2 diabetes (T2DM) was induced by giving high-fat diet (HFD) for 3 weeks followed by a single streptozotocin (STZ) injection (40 mg/kg, i.p., once dose) followed by injection of 9 doses of rotenone every 48 ± 2 h for induction of PD. Met and/or Sema were administered to DM+PD via gastric gavage once daily for 4 weeks. In comparison with the DM+PD group, Met and/or Sem significantly lowered blood glucose levels, HOMA-IR, HbA1C, cholesterol, triglycerides, and LDL with significantly increased insulin and HDL levels. In addition, there was enhanced brain antioxidant status with lower oxidative-inflammatory stress biomarkers associated with improved rat cognitive, locomotor, and olfactory functions. A significant downregulation of caspase 3 and GFAP with concomitant upregulation of NRF2 protein expressions were observed in treated groups. Overall, co-treatment with Met and Sem elicited more efficacy than that of the individual regimen. When combined, the results of this study have demonstrated for the first time that Met and Sem work in concert to create neuroprotection in PD model of male diabetic rats compared to when taken separately. The study’s findings indicate that Met and/or Sem have a restorative effect on T2DM and PD-induced changes in neurobehavioral and biochemical/molecular indices ascribed to the improvement of endogenous antioxidant systems, decreased lipid peroxidation, suppression of oxidative/inflammatory stress, and—most importantly—regulation of Nrf2 and caspase 3.

**Graphical abstract:**

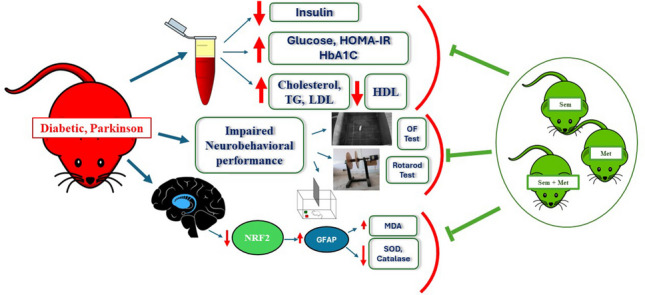

## Introduction

Among metabolic disorders, diabetes mellitus (DM) might be considered the most common, rapidly increasing, and hazardous medical issue worldwide. Poorly managed or uncontrolled DM can lead to major complications such as neuropathy, nephropathy, and retinopathy with consequent serious morbidity (Narayan et al. 2000; Zatalia and Sanusi 2013). Nearly 50% of diabetic individuals have diabetic neuropathy (Abbott et al. 2011; Selvarajah et al. 2011) probably due to deficiency of insulin action in the brain besides damage induced by persistent hyperglycemia (Brands et al. 2004). Hyperglycemia induces cognitive damage by increasing neuronal brain inflammation and oxidative stress (Brands et al. 2004). Numerous studies revealed that DM increases the risk of neurodegenerative diseases like Alzheimer’s and PD. The brain region most affected by changes linked to DM is the hippocampus (Foghi and Ahmadpour 2014).

PD affects more than 6 million individuals globally; by 2040, that number is expected to reach more than 12 million (Agostini et al. 2022). One of the hypothesized mechanisms of PD is insulin desensitization. Clinical statistics show that 8–30% of PD patients had DM, a much higher percentage than the age-matched control group (Zhang et al. 2018).

Tremor, stiffness, bradykinesia, and postural instability are some PD motor symptoms, as well non-motor symptoms including constipation, postural hypotension, rapid eye movement sleep behavior disorder, apathy, and dementia (Tofaris 2022). Both the existence of pathogenic neuronal alpha-synuclein (α-syn) aggregates, also known as Lewy bodies and Lewy neurites, and loss of dopaminergic neurons in the substantia nigra (SN) pars compacta are hallmarks of PD (Berge and Ulusoy 2022). α-syn may stimulate microglia, which then releases proinflammatory cytokines that may block downstream insulin transmission and cause neuronal insulin resistance.

Reduced energy expenditure, gene expression, cell repair, and autophagy can be the outcome of insulin desensitization because of insulin's critical function as a growth factor in the brain (Zhang et al. 2022). Recently, Met is recommended as a viable option for a novel PD therapy among the several anti-diabetic medications currently on the market (Biosa et al. 2018). Met is one of the first-line oral hypoglycemic drugs that act through enhancing peripheral glucose absorption, suppressing hepatic gluconeogenesis and boosting insulin sensitivity (Oliveira et al. 2022). Several studies have recently suggested its possible role in neurodegenerative disorders such as Alzheimer’s, amnestic moderate cognitive impairment, and PD (Markowicz-Piasecka et al. 2017).

In T2DM, glucagon-like peptide-1 (GLP-1) analogs act through GLP-1receptor which promote insulin signaling and glucose control. GLP-1 analogs can pass the blood brain barrier and act on GLP-1 receptor located in the frontal cortex, hypothalamus, thalamus, hippocampus, cerebellum, and substantia nigra of the brain affecting several biological pathways in the central nervous system (CNS), including neuroinflammation, mitochondrial function, and cell proliferation (Liu et al. 2022). The longer-acting GLP-1 receptor agonist; Sem (C187H291N45O59) shares 94% structural similarities with natural GLP-1. Sem has been studied to improve cognitive performance and lower body weight, steatosis, and hyperglycemia in neurodegenerative disorders (Mahapatra et al. 2022) besides its neuroprotective effect in animal models of PD, epilepsy, and ischemic stroke. The scope of our study is to provide further insights into the neuroprotective effect of Met and/or Sem on the PD model in male diabetic rats and the possible underlying mechanism.

## Materials and methods

### Animals and experimental groups

Forty adult male Wister albino rats of local strain, weighing 150 ± 20g were purchased from the national research center animal house (Cairo, Egypt). Rats were housed in an animal house at the Faculty of Medicine, Menoufia University, Egypt (humidity 60 ± 10%, temperature 25 ± 2 °C, and 12 h light/dark cycle). Rats have unrestricted access to food and water during the experiment. The Local Ethics Committee granted approval for the experimental protocol, which strictly followed the ARRIVE guidelines for reporting in vivo experiments and the use of laboratory animals. After 7 days of acclimatization, rats were randomly divided into 5 experimental groups (*n* = 8):

1. Control (Cont.) group: Rats had free access to standard laboratory diet and water. The vehicle (sterile saline solution 0.9% NaCl) was administered via gastric gavage across various of the study.
2. Non-treated diabetic, Parkinson (DM+PD) group: T2DM was induced by giving HFD for 3 weeks followed by a single STZ (Sigma Chemical Company, USA) injection (40 mg/kg body weight, i.p., dissolved in cold 0.01M citrate buffer, pH 4.5). Periodic measurement of blood glucose (days 0, 3, and 7) was carried out to confirm induction of DM (Suman et al. 2016). PD was induced by injection of 9 doses of rotenone (Sigma Chemical Company, USA) (1 mg/kg, S.C.) every 48 ± 2 h (Zaitone et al. 2019).
3. Sem-treated diabetic, Parkinson (Sem) group: Sem in a dose of (50 nmol/kg) was administered to **DM+PD** via gastric gavage once daily for 4 weeks (Melander et al. 2023).
4. Met-treated diabetic, Parkinson (Met) group: Met (CID company, Cairo, Egypt) (250 mg/kg) was administered to **DM+PD** via gastric gavage once daily for 4 weeks.
5. Combined Sem/Met-treated diabetic, Parkinson (Sem + Met) group: Met and Sem were administered to **DM+PD** via gastric gavage once daily for 4 weeks

Following a 4-week experimental period, neurological and behavioral evaluation tests were performed, and blood samples were subsequently taken immediately before scarification. The rats were then sacrificed by an intraperitoneal injection of ketamine (50 mg/kg) and xylazine (10 mg/kg) mixture and brains were meticulously dissected. Rats’ left cerebral hemispheres’ basal ganglia were extracted and the striatal tissues were homogenized for biochemical studies. The right cerebral hemispheres were kept 10% formalin for histopathological and immunohistochemical evaluation.

### Body weight

The body weight of all studied groups at the start, after 2 weeks of HFD, and at the end of the study.

### Biochemical studies

At the end of the experiment, rats were fasted overnight. Blood samples were collected by a tiny, heparinized micro-capillary tube inserted into the medial epicanthus of the rats’ eyes to draw blood from the retro-orbital venous plexus for measuring HbA_1_C by specific kits (Bio Diagnostic Company, Egypt). Other blood samples were collected in a clean graduated tube, centrifuged for 15 min at 3000 rpm, and then stored at 37 °C for 45 min. Fasting blood glucose level; serum lipid profile (serum total cholesterol, triglycerides (TG); low-density lipoprotein (LDL); and high-density lipoprotein (HDL)) (Bio diagnostic Company, Egypt) was colorimetrically measured according to the method of Watts and Caraway (Caraway and Watts 1970). Serum insulin level was measured (Crystal Chem, USA) according to manufacturer instruction. Insulin resistance (IR) was measured using homeostatic model assessment for insulin resistance (HOMA-IR). The calculation was performed according to the following equation (Elgarawany et al. 2023):

$$\mathrm{HOMA}-\mathrm{IR}\;=\;\frac{\mathrm{Fasting}\;\mathrm{insulin}\;(\mathrm{uIU}/\mathrm{ml})\times\mathrm{Fasting}\;\mathrm{blood}\;\mathrm{glucos}\;(\mathrm{mmol}/\mathrm l)}{22.5}$$

Following rats’ sacrifice, left hemisphere was dissected, and the basal ganglia were homogenized to estimate dopamine level by double-antibody sandwich ELISA kit (Sunred Company, Shanghai, China). Brain-derived neurotrophic factor; brain-derived neurotrophic factor (BDNF), C-X3-C motif chemokine ligand 1 (CX3Cl1) (ELK Biotechnology company, Wuhan, China), tumor necrosis factor alpha (TNF-α; ELK Biotechnology company, Wuhan, China) and interleukin 10 (IL-10; ELK Biotechnology company, Wuhan, China) levels were assessed, respectively.

### Neurological and behavioral evaluation tests

#### Open field (OF) test

The test was performed according to the protocols of Aksu et al. (Aksu et al. 2012) for the assessment of locomotor activity and degree of anxiety. The test chamber is white, square shaped, with dimensions of (1 m × 1 m × 50 cm). Its floor is divided into squares (20 cm × 20 cm/each). Rats’ movements were recorded for 5 min by digital camera. The squares traversed, center crossing, rearing, and grooming were observed and calculated. The test chamber was wiped between trials with a 70% ethyl alcohol solution.

#### Rotarod test

A stationary Rotarod test (Harvard Apparatus, UK) was carried out to assess the motor coordination of the studied rats. The device has a revolving spindle, a power supply to spin it, and grids underneath the roller so the rat can fall off it safely. All animals were pre-trained on the rotarod apparatus for 5 days to reach a stable performance. At a speed of 15 rpm, the rotarod spun for 120 s. If the rat fell during the 120-s test, the experiment was considered over (Can et al. 2012).

#### Olfactory preference test

Small portions of either water or a novel smell (vanilla) were given to the rats at the same time. Each odor is given a smelling time of 3 min (Tillerson et al. 2006).

### ELISA studies

Following the sacrifice of the rats, the brain was taken one hemisphere was dissected, and the basal ganglia was homogenized to estimate CX3Cl1 levels by specific ELISA kits, BDNF, and dopamine level by double-antibody sandwich ELISA kit (Sunred Company, Shanghai, China) following the manufacturer’s instructions. Using the relevant rat ELISA kits TNF-α and IL-10 levels were assessed, respectively.

### Oxidative stress markers in the basal ganglia

Malondialdehyde (MDA) and catalase (CAT) activities were determined using specific colorimetric kits (Biodiagnostic Company, Egypt), following the manufacturer’s instructions.

### Histopathological studies

Brain specimens were fixed in 10% neutral buffer formalin, trimmed, washed in water, dehydrated in ascending grades of ethyl alcohol, cleared in xylene, and embedded in paraffin. Thin sections (4–6µ) were processed and stained with Hematoxylin and Eosin (H&E) stain (Bancroft 2008). Detected lesions in the brain tissues were scored through determination of the percentage of the lesion frequency per five non-repeated randomly selected microscopic fields (40×) per animal (thirty fields/organ/group) using the following score system; 0 = absence of the lesion in all rats of the group, 1=1–10%, 2=11–25%, 3=26–50%, 4=51–75%, 5 = over 75% and are summarized in (Table 4).

### Immunohistochemical (IHC) studies

Paraffin sections were mounted on positively charged slides by using avidinbiotin-peroxidase complex (ABC) method. Sections from each group were incubated with the required primary antibodies; polyclonal rabbit caspase-3 antibody (1:700; Service bio (GB11532)), monoclonal mouse (Glial fibrillary acidic protein) GFAP antibody (1:800; Service bio (GB12090)), and polyclonal rabbit nuclear erythroid 2-related factor 2 (NRF2) (1:500; Novusbio (NBP1-32822)). ABC method reagents were added (Vectastain ABC-HRP kit, Vector laboratories) thereafter. Marker expression was labeled with peroxidase and colored with diaminobenzidine (DAB, produced by Sigma) to detect antigen-antibody complex. Negative controls were included using non-immune serum in place of the primary or secondary antibodies. IHC-stained sections were examined via using Olympus microscope (BX-53). Scoring of immunohistochemistry results by determination of reaction area percentage in 10 microscopic fields using image J 1.53t, Wayne Rasband and contributors, National Institutes of Health, USA.

### Statistical analysis

All data were expressed as the mean ± standard deviation (SD). The data were analyzed using SPSS program version 22.0 (SPSS Inc., Chicago, IL, USA). One-way ANOVA followed by post hoc Tukey’s multiple comparison test. *p* value *≤ 0.05* was considered statistically significant.

## Results

### Body weight

According to Table 1, there was no discernible difference in body weight between the groups studied at the start of the study or even after two weeks of a high-fat diet. A significant decline was observed at the end of the study in DM+PD group compared to Cont. group (*P* < 0.05). Sem, Met, and Sem+Met groups had statistically significant lower body weights (*P* < 0.05) compared to Cont. and DM+PD groups.

**Table 1:** Body weight of all studied groups at the start, after 2 weeks of HFD, and at the end of the study

	Cont.	DM+PD	Sem	Met	Sem + Met
Start	131.5 ± 8.9	136.2 ± 7.4	129.5 ± 9.2	133.6 ± 8.2	132.8 ± 9.9
2ws	192.6 ± 9.5	191.5 ± 9.4	193.5 ± 9.5	193 ± 9.5	194.4 ± 9.9
End	206.8 ± 16.2	176.3 ± 7.4^*^	143 ± 7.9^*#^	156.7 ± 8.9^*#$^	129.9 ± 5.4^*#$€^

Data are expressed as mean ± SD (*n* = 6). Multiple comparisons were done using one-way ANOVA followed by Tukey’s as a post-ANOVA test: *p < 0.05, vs control; ^*#*^*p* < 0.05, vs DM+PD; ^*$*^*p* < 0.05, vs Sem; ^*€*^*p* < 0.05, vs Met

### Biochemical studies

When compared to Cont. group, rats of DM + PD group, demonstrated markedly lower blood insulin (µIU/ml) and HDL (mg/dl) levels (*P* < 0.05) along with a significant increase (*P* < 0.05) in serum glucose (mg/dl), HBA1C (%), HOMA-IR, total cholesterol (mg/dl), LDL (mg/dl), and TG (mg/dl) levels. An improvement in glycemic state and lipid profile was noted in treated groups, with a more notable effect observed Sem + Met group (*P* < 0.05) compared to each of Sem and Met groups (Table 2).

**Table 2:** Serum glucose, insulin, HOMA-IR, HbA1c, cholesterol, TG, LDL, and HDL in all studied groups

Groups	Cont.	DM+PD	Sem	Met	Sem+Met
Glucose (mg/dl)	87.97 ± 5.03	288.79 ± 40.28 *	126.96 ± 5.95*# €	157.77 ± 8.77*# $	102.78 ± 6.81 # $ €
Insulin (µIU/ml)	11.87 ± 1.22	6.49 ± 0.82*	9.56 ± 0.56*#€	8.16 ± 0.43*#$	11.11 ± 1.09 #$€
HOMA‑IR	2.58 ± 0.35	4.6 ± 0.78*	2.99 ± 0.2#	3.18 ± 0.22*#	2.8 ± 0.16 #
HA_1_C	2.33 ± 0.5	5.9 ± 0.55*	3.64 ± 0.76*#€	4.65 ± 0.5*#$	2.52 ± 0.59 #$€
Cholesterol (mg/dl)	61.82 ± 4.21	110.02 ± 7.28*	89.05 ± 3.73*#€	99.68 ± 3.65*#$	75.01 ± 4.62*#$€
TG (mg/dl)	81.71 ± 4.69	138.82 ± 9.04*	99.96 ± 4.06*#€	113.64 ± 5.05*#$	91.08 ± 5.58*#$€
LDL (mg/dl)	81.34 ± 4.28	138.99 ± 5.37*	102.67 ± 4.29*#€	114.38 ± 4.59*#$	94.53 ± 3.77*#$€
HDL (mg/dl)	38.44 ± 2.11	23.81 ± 2.87*	29.61 ± 1.55*#€	26.6 ± 1.55*#$	33.36 ± 2.33*#$€

Data are expressed as mean ± SD. (*n* = 10). Multiple comparisons were done using one-way ANOVA followed by Tukey’s as a post-ANOVA test: *p < 0.05, vs control; ^*#*^*p* < 0.05, versus DM+PD; ^*$*^*p* < 0.05, vs Sem; ^*€*^*p* < 0.05, vs Met

### Neurological and behavioral evaluation tests

#### Open field (OF) test

As shown in (Table 3), there was a significant increase (*P* < 0.05) in the latent period, rearing, and grooming with concomitant decrease (*P* < 0.05) in crossed squares in DM+PD group compared with Cont group. Met group showed a substantial decrease (*P* < 0.05) in rearing and grooming with accompanying increase (*P* < 0.05) in crossed squares compared with DM+PD group. Sem group showed a significant rise (*P* < 0.05) in crossing squares and with concurrent decrease (*P* < 0.05) in latent period, rearing, and grooming compared to DM+PD and Met groups. There was a significant (*P* < 0.05) drop in rearing and grooming and a commensurate increase (*P* < 0.05) in crossing squares between the Sem + Met group and the DM+PD, Met, and Sem groups.

**Table 3:** Neurobehavioral tests (rat performance in open field test, rotarod test, and olfactory preference test) in all studied groups

	Cont.	DM+PD	Sem	Met	Sem + Met
Latent period	35.1 ± 9.1	94.7 ± 22.8^*^	70.5 ± 7.7^*#^	86.8 ± 5.6^*$^	60.9 ± 9^*#$^
Center crossing	46.3 ± 9.8	43.3 ± 9.4	42.7 ± 9	43.5 ± 7.7	45.3 ± 9
Crossed squares	72.4 ± 7.3	25.7 ± 3.2^*^	45.1 ± 3.3^*#^	35.3 ± 3.9^*#$^	55.4 ± 4.1^*#$€^
Rearing	1.9 ± 0.3	10.4 ± 0.8^*^	6.9 ± 0.7^*#^	8.9 ± 0.6^*#$^	4.9 ± 0.6^*#$€^
Grooming	2.2 ± 0.6	12.3 ± 0.8^*^	8 ± 0.6^*#^	9.9 ± 0.6^*#$^	4.9 ± 0.7^*#$€^
Latency to fall	117.5 ± 5.4	39.8 ± 6.6^*^	84.7 ± 3.9^*#^	79.5 ± 4^*#^	97.4 ± 5.7^*#$€^
Duration of olfaction	146.5 ± 10.4	36.8 ± 9.7^*^	84.1 ± 4.7^*#^	76.6 ± 3.9^*#$^	106.6 ± 5.3^*#$€^

Data are expressed as mean ± SD (*n* = 6). Multiple comparisons were done using one-way ANOVA followed by Tukey’s as a post-ANOVA test: *p < 0.05, vs control; ^*#*^*p* < 0.05, vs DM+PD; ^*$*^*p* < 0.05, vs Sem; ^*€*^*p* < 0.05, vs Met

#### Rotarod test

DM+PD group latency to fall was significantly lower (*P* < 0.05) than that of Cont. group. There was a discernible increase (*P* < 0.05) in latency to fall in Sem + Met group compared to DM+PD group which was more noticeable (*P* < 0.05) than in Sem and Met groups separately (Table 3).

#### Olfactory preference test

There was a significant decline (*P* < 0.05) in olfactory duration in DM+PD group compared to Cont. group. Met, Sem and Sem + Met groups showed a significant increase (*P* < 0.05) in olfactory duration when compared with DM+PD group with more incredible effect observed in Sem + Met group (Table 3).

### ELISA studies

**Fig. 1:**
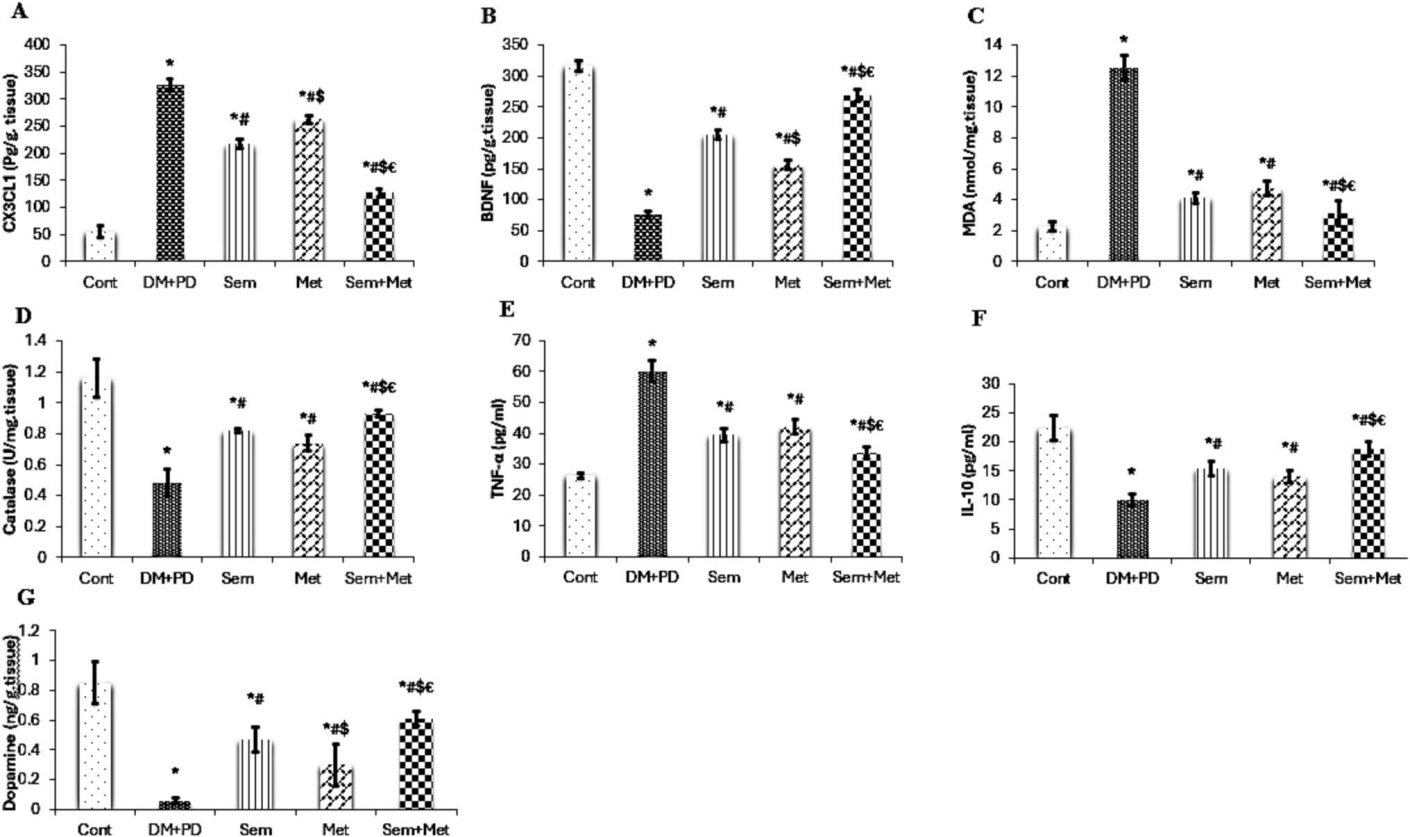
ELISA studies of CX3CL1, BDNF, TNF-α, IL-10, and dopamine and a colorimetric assay of MDA and catalase of the different studied groups. Data are expressed as mean ± SD (*n* = 6). Multiple comparisons were done using one-way ANOVA followed by Tukey’s as a post-ANOVA test: *p < 0.05, vs control; #*p* < 0.05, versus DM+PD; $*p* < 0.05, vs Sem; €*p* < 0.05, vs Met

CX3CL1 level was significantly higher in DM+PD group compared to Cont. group (Fig. 1A). A noteworthy drop in each of the three treated groups compared to the diseased group was detected (Fig. 1A). However, Sem + Met group experienced the most noticeable effect. CX3CL1 level was much lower in Sem compared to Met group (Fig. 1A). Compared to Cont. group, BDNF was significantly decreased in DM+PD group (Fig. 1B). A remarkable rise was observed in Sem + Met group compared to the two other treated groups (Fig. 1B). A more incredible effect was observed in Sem compared to Met group (Fig. 1B). There was an apparent decrease in dopamine levels in the DM+PD group compared to Cont. group (Fig. 1G). A matching increase was detected in Sem, Met, and Sem + Met groups with a more apparent effect observed in Sem + Met group (Fig. 1G). TNF-α level (pg/ml) in DM+PD group was significantly higher than that of Cont. group (Fig. 1E). Sem, Met, and Sem + Met groups showed a significant decrease in TNF-α levels compared to DM+PD group with more remarkable effect in Sem + Met group compared to each of Sem and Met groups (Fig. 1E). IL-10 level (pg/ml) was significantly decreased in DM + PD group compared to Cont. group (Fig. 1F). Sem, Met, and Sem + Met groups showed a statistically significant rise in IL-10 level compared to DM+PD group (Fig. 1F). Compared to the Sem and Met groups individually, the IL-10 increase was more pronounced in the Sem + Met group (Fig. 1F).

### Oxidative stress markers in the basal ganglia

There was a significant increase in MDA level (nmol/mg tissue) in DM+PD group compared with Cont. group (Fig. 1C). A significant decline in MDA level was observed in Sem, Met, and Sem + Met groups compared with DM+PD group (Fig. 1C). Sem + Met group showed a more notable decline in MDA level than did the Sem and Met groups separately (Fig. 1C). CAT level (U/mg tissue) in DM+PD group was significantly lower than in Cont. group (Fig. 1D). Sem, Met, and Sem + Met groups showed a significant increase in CAT levels as compared to DM+PD group (Fig. 1D). There was a discernible rise in CAT levels in Sem + Met group compared to other treated groups (Fig. 1D).

### Histopathological studies

**Fig. 2:**
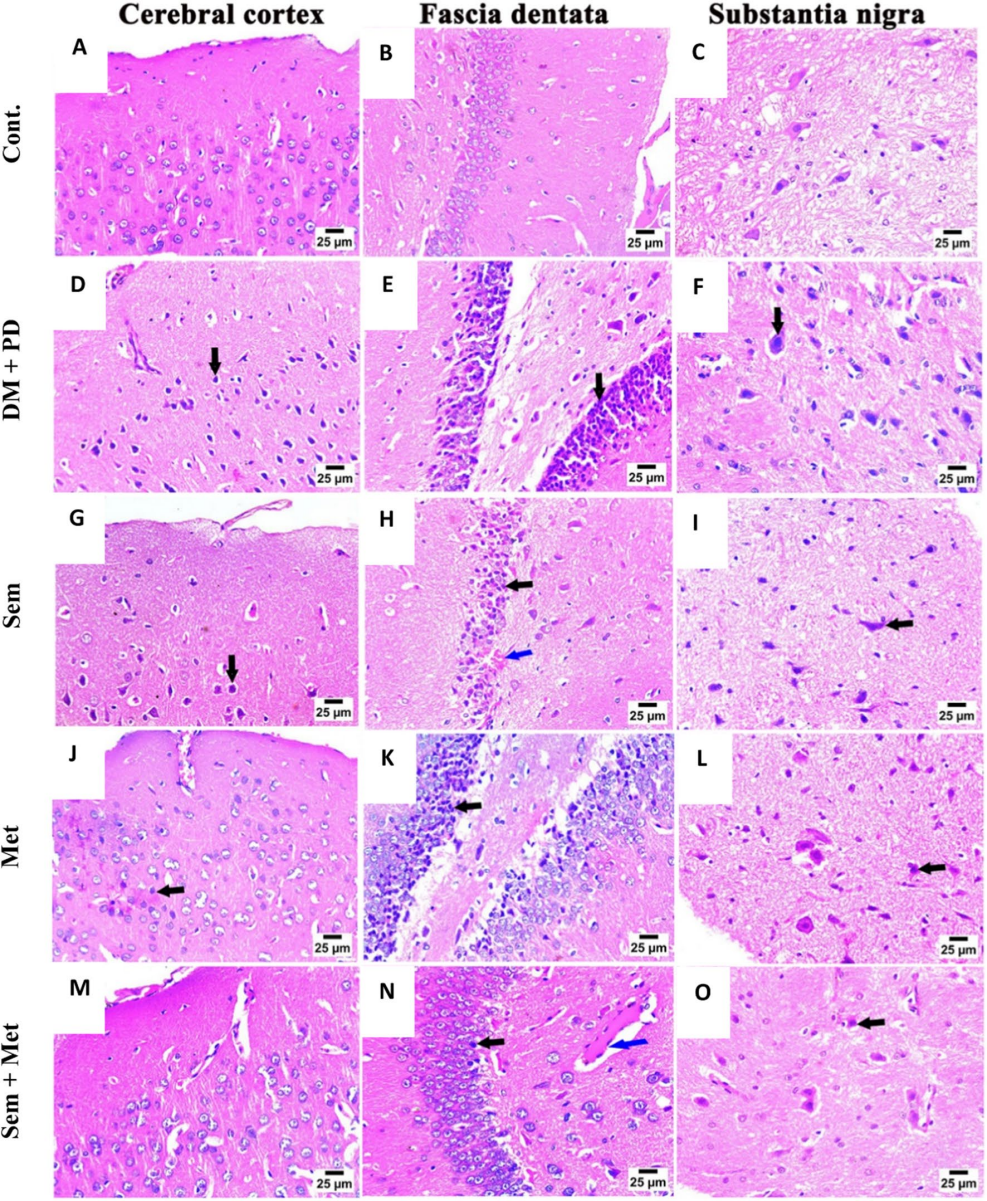
Photomicrograph of the cerebral cortex, fascia dentata, and substantia nigra of brain tissues of the different studied groups (hematoxylin and eosin stain). Cont. group showing normal histological structured of the cerebral cortex (**A**), fascia dentate (**B**) in the hippocampus and substantia nigra (**C**). DM+PD showing severe shrunken and degenerated neurons with pyknotic nuclei (arrow) in the cerebral cortex (**D**) with severe nuclear pyknosis in neurons (arrow) of fascia dentata (**E**) and substantia nigra (**F**). Sem group showing moderate shrunken and degenerated neurons with pyknotic nuclei (arrow) in the cerebral cortex (**G**) with severe nuclear pyknosis in neurons (black arrow) with hemorrhage (blue arrow) in fascia dentate (**H**) and substantia nigra (**I**). Met group showing mild nuclear pyknosis in some neurons (arrow) of the cerebral cortex (**J**) with moderate nuclear pyknosis (arrow) in neurons of fascia dentata (**K**) and substantia nigra (**L**). Sem + Met group showing normal histological structure of cerebral cortex (**M**) with mild nuclear pyknosis (black arrow) in neurons and presence of congestion of minute blood vessels with perivascular edema (blue arrow) of fascia dentata (**N**) and mild nuclear pyknosis (arrow) in some neurons of substantia nigra (**O**)

Histopathological examination of Cont. brain tissue showed normal histological features with no discernible variations (Fig. 2A–C). DM+PD group showed sever shrunken, degenerated neurons with pyknotic nuclei, deposition of Lewis body within the substantia nigra associated with hemorrhaging and empty spaces indicating significant neuronal loss (Fig. 2D–F). Mild to moderate shrunken and degenerated neurons with pyknotic nuclei in cerebral cortex, fascia dentata, and substantia nigra were detected in Sem and Met groups (Fig. 2G–L). Sem + Met group displayed significantly reduced severity of the neurodegenerative changes with only a few scattered pyknotic neurons with perineural vacuolations and congestion of minute blood vessels with perivascular edema in neurons of fascia dentata and substantia nigra (Fig. 2M–O). Areduction was seen in the lesion severity was shown in Sem, Met, and Sem + Met groups compared to the DM + PD (Table 4).

**Table 4:** Lesion scoring in the brain tissues of all studied groups

	Cont.	DM+PD	Sem	Met	Sem + Met
Hemorrhages	0	2	2	2	0
Neuronal pyknosis	0	4	1	1	0
Perineural vacuolations	0	3	1	1	1
Microgliosis	0	3	1	1	1
Reactive astrogliosis	0	3	2	2	0
Cerebral congestion	0	2	1	1	1
Meningeal congestion	0	2	1	1	1

The score system was designed as follows: score 0, absence of the lesion in all rats of the group; score 1, 1–10%; score 2, 11–25%; score 3, 26–50%; score 4, 51–75%; score 5, over 75%

### Immunohistochemical (IHC) studies

**Fig. 3:**
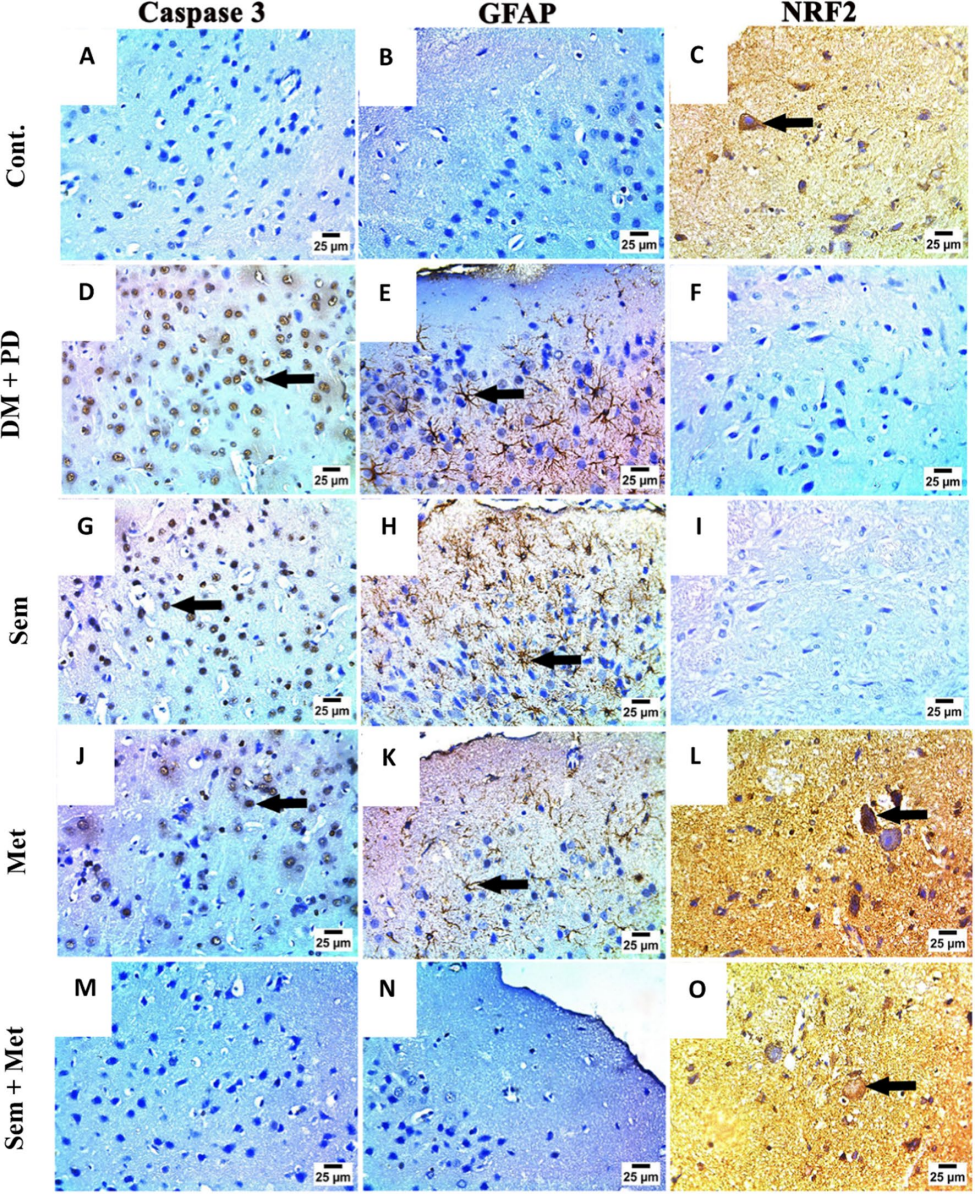
Photomicrograph of Caspase 3, GFAP, and NRF2 immunohistochemistry in the striatum neurons of the different studied groups. Photomicrograph of caspase 3 immunohistochemistry of control group (**A**) showing negative expression, DM + PD group (**D**) showing severe positive expression (arrow), Sem group (**G**) showing severe positive expression (arrow), Met group (**J**) showing moderate positive expression (arrow), combined Sem + Met group (**M**) showing negative expression. Photomicrograph of *GFAP* Immunohistochemistry in the cerebral cortex neurons of: Cont group (**B**) showing negative expression, DM + PD group (**E**) showing severe positive expression (arrow), Sem group (**H)** showing severe positive expression (arrow), Met group (**K**) showing mild positive expression (arrow), Sem + Met group (**N**) showing negative expression. Photomicrograph of *NRF2* (**C**) immunohistochemistry in the neuronal cytoplasm of substantia nigra of Cont. group (**C**) showing severe positive expression (arrow), DM + PD group (**F**) showing negative expression, Sem group (**I**) showing negative expression, Met group (**L**) showing severe positive expression in some neuronal cytoplasm (arrow), Sem + Met group (**O**) showing severe positive expression (arrow) in neuronal cytoplasm. (IHC-Peroxidase-DAB)

Immunostaining for striatal caspase 3 showed negative expression in Cont. group (Fig. 3A). Nonetheless, there was substantial expression in the DM + PD (Fig. 3D) and Sem (Fig. 3G) groups. The expression within Met group was mild (Fig. 3J). Conversely, the Sem + Met group displayed a negative expression (Fig. 3M). GFAB expressions revealed negative expression in Cont group (Fig. 3B). However, DM + PD **(**Fig. 3E**)** and Sem **(**Fig. 3H**)** groups showed strong expression. Met group **(**Fig. 3K**)** displayed mild expression; in contrast, Sem + Met **(**Fig. 3N**)** group exhibited negative expressions. NRF2 immunostaining revealed strong cytoplasmic staining in Cont. group in the substantia nigra region (Fig. 3C). However, DM + PD **(**Fig. 3F**)** and Sem **(**Fig. 3I**)** groups showed negative staining. Met **(**Fig. 3L**)** and Sem + Met **(**Fig. 3O**)** showed strong staining as Cont. one.

## Discussion

People with diabetes are much more prone to suffering from mental health problems and have their brain function affected. When compared to the effects of each medication alone, the results of this investigation demonstrated for the first time that Met and Sem work in concert to create neuroprotection in PD model of male diabetic rats. The study’s findings suggest that Met and/or Sem counteract T2DM and PD-induced changes in neurobehavioral and biochemical/molecular indices by boosting endogenous antioxidant systems, lowering lipid peroxidation, suppressing oxidative/inflammatory stress, and—most importantly, regulating Nrf2 and caspase 3.

STZ dramatically raises the dopaminergic neurons’ susceptibility to degeneration. The rotarod test of motor coordination, and the open field observation of spontaneous movement and exploration all demonstrated the obvious motor deficits caused by rotenone. The test of olfactory preference was indicated to measure olfactory performance in PD. The current investigation demonstrated that treatment with Sem and/or Met can prevent the overactivity of the pro-inflammatory axis that STZ generates, which raises oxidative stress, neuroinflammation, and neurodegeneration. The activation of the transcription factor Nrf2 in astrocytes through the GFAP promoter, which synchronizes the induction of antioxidant defense and provides protection to nearby neurons, may account for the neuroprotective action of Sem and/or Met. DM is a metabolic disease that has become a major global public health concern. By 2040, there will be 642 million adult diabetics worldwide, according to estimates (Hao et al. 2021). Diabetes frequently causes a wide range of chronic side effects such as peripheral neuropathy, renal disease, retinopathy, and cardiovascular disease beside brain problems (McCrimmon et al. 2012).

Increasing evidence indicates that diabetes is a risk factor for an increased incidence of PD (Zhao et al. 2023; Foltynie and Athauda 2020; Cheong et al. 2020). High levels of glucose in the cerebrospinal fluid (CSF) of diabetic rats may intensify the impact of a subthreshold dosage of the neurotoxin 6-hydroxydopamine (6-OHDA) on the onset of motor abnormalities and damage on the nigrostriatal dopaminergic neuronal pathway’s damage (Zhao et al. 2023). Insulin resistance in patients with PD is associated with accelerated disease progression, increased severity of movement disorders, and an increased risk of PD (Tamtaji et al. 2019). There is currently no clinically viable disease-modifying strategy available for PD. The commonly utilized therapy alternatives only offer little symptomatic alleviation (Wang et al. 2024).

Insulin signaling abnormalities are an established hallmark in older adults with pre-diabetes/T2DM and have also been observed in PD (Banks et al. 2012; Hoyer 2004). Given the biological similarities between DM and neurodegenerative disorders (PD), anti-diabetic medications hold a significant promise as a treatment approach. Met is an insulin-sensitizing biguanide commonly used as first-line oral regimen in patients with T2DM particularly who are overweight, in the absence of contraindications (Inzucchi et al. 2015). Met potential for treating neurodegenerative illnesses has recently attracted interest (Markowicz-Piasecka et al. 2017). Long-term Met use (> 2 years) significantly reduced the chance of acquiring neurological illnesses in 5528 individuals with T2DM, including dementia, AD, PD, Huntington’s disease, and MCI (Shi et al. 2019). The results of several experimental and clinical studies indicate that Met improves cognitive function and protects the brain against the oxidative imbalance imposed by diabetes (Correia et al. 2008) as indicated in our study.

The only GLP-1 receptor agonist accessible as an oral medication is Sem, a long-lasting agonist that is being used as a once-weekly subcutaneous injection. Numerous beneficial benefits have been linked to GLP-1 signaling, including the induction of anti-inflammatory signaling, the reduction of oxidative stress as confirmed in our study. Besides, the enhancement of gene transcription, and the regulation of autophagy have been studied (Calsolaro and Edison 2015; Grieco et al. 2019). Targeting GLP-1 signaling represents a promising neuroprotective and potentially disease-modifying strategy for PD (Brauer et al. 2020).

Neuroinflammation may be a major factor in the dopaminergic cell loss as well as the presence of activated microglia and astrocytes in PD (Ryu et al. 2020). Astrocytes are crucial for preserving the homeostasis and health of neurons (Liddelow and Barres 2017). They are involved in inflammatory responses during neurodegenerative diseases (Gorshkov et al. 2018). The assessment of the induction of astrocyte reactivity after dopaminergic cell loss and ageing was conducted using the standard astrocyte gene marker, GFAP (Ryu et al. 2020). Met and/or Sem decreased GFAP immunostaining in the striatal neurons (SN), a sign of astrocyte activation, compared to DM+PD group, which exhibited high expression. In agreement with our study, Met-treated group showed reduced astrocyte activity compared to the vehicle-treated group in the 6-OHDA-lesioned striatum with improved motor impairments even though dopaminergic cell death was not prevented (Ryu et al. 2020). Moreover, Met remedy substantially inhibited age-induced GFAP activation (Ryu et al. 2020). To lessen the disruption of the BBB in mice following an ischemic stroke, Sem inhibits the development of C3d+/GFAP+ astrocytes (Zhang et al. 2022). Combination therapy with Sem and rosiglitazone was studied for diabetic retinopathy in rodent animals by decreasing the GFAP expression and inhibiting oxidative stress (Yang et al. 2021).

BDNF is a neurotrophins essential for neuronal development and survival, synaptic plasticity, and cognitive function (Ryu et al. 2020). Dysregulation of BDNF signaling is involved in several neurodegenerative disorders, including PD. It is produced mainly centrally alongside peripheral tissue (Rozanska et al. 2020). Activated immune cells, adipose tissues, liver, skeletal muscle, and endothelial cells are all known to express BDNF in addition to its availability in blood and serum (Rozanska et al. 2020). BDNF is also named “metabokine” because of its effects on glycemia, lipid profile and energy homeostasis beside its role in metabolic control, especially glucose metabolism and insulin resistance (Rozanska et al. 2020). Obesity, T2DM, and PD are all linked to altered BDNF levels (Suwa et al. 2006) as indicated in our study.

Insulin resistance appears to affect and be related to the amount and function of BDNF in diabetes as T2DM patients demonstrate a drop of BDNF in their circulation (Chan et al. 2019) in accordance with our study in rat group. Met and/or Sem therapy elevated BDNF level suppressed in the diseased group. Similarly, BDNF signaling pathways were induced by Met treatment on the 6-OHDA-lesioned side of the striatum (Ryu et al. 2020). Through the CREB/BDNF axis, Sem increases remyelination and overcomes demyelination, therefore can amend experimental autoimmune encephalomyelitis (EAE)-induced multiple sclerosis in mice (Sadek et al. 2023).

Met alleviated inflammation in the DM+PD group by mitigating serum levels of pro-inflammatory TNF-α and anti-inflammatory IL-10, which is consistent with other research that shown Met enhancing memory and learning in APP/PS1 through improved neurogenesis and decreased inflammation (Ou et al. 2018; Saffari et al. 2020). Evidence implies that Met has in vivo anti-inflammatory properties in both MPTP-induced damage of the nigrostriatal dopaminergic system (Ismaiel et al. 2016) and in STZ-induced diabetic mice (Oliveira et al. 2016).

When high glucose levels cause destabilization of homeostasis, the ratio of pro- to anti-inflammatory cytokines usually shifts. This includes activation of chemokine (chemotactic cytokine) CX3CL1 (fractalkine, neurotactin) (Szukiewicz et al. 2018; Szukiewicz et al. 2013), which is largely responsible for the pathogenesis and pathophysiology of diabetes (Yao et al. 2014; Das and Mukhopadhyay 2011). TNF-α induces the expression of CX3CL1 in rat aortic smooth muscle cells through the NF-κB pathway (Sung et al. 2010). Deficiency of the CX3CL1 receptor employs a protective effect on glucose intolerance and insulin resistance (Shah et al. 2015). Neurons produce CX3CL1 which communicates via its specific receptor found on microglial cells (Subbarayan et al. 2022). Numerous studies have demonstrated the neuroprotective properties of CX3CL1, which is thought to be primarily involved in reducing the proinflammatory response within the CNS. However, it seems to be encouraging neurodegeneration in certain situations (Subbarayan et al. 2022).

As Met reduces the synthesis of TNF-α, it may trigger an autoregulatory mechanism that influences the CX3CR1 expression (Matsumiya et al. 2010; D’Haese et al. 2010) in agreement with our results. Met therapeutic effects in lowering hyperglycemia in obese people, those with T2DM, or those with impaired glucose tolerance may be mostly linked to decreased TNF-α and CX3CL1 production (Szukiewicz et al. 2018).

Several reports have implicated Nrf2 as the main transcription factor of antioxidative stress (Zhang et al. 2018). The core role of Nrf2 is established through interaction with antioxidant response elements to stimulate the expression of several downstream targets involved in cellular protection (Ebokaiwe et al. 2020). In agreement with our study, a significant decrease of Nrf2 expression in STZ-diabetes rats was detected as consequent to devastating oxidative stress following hyperglycemia. In response to free radical exposure, Nrf2 dissociates from Keap-1, its cytosolic inhibitor, and moves to the nucleus, where it joins the antioxidant response element, the promoter area of numerous phase II enzymes (Gao et al. 2020).

Administration of Met and/or Sem restored Nrf2 expression to basal levels, which could be one of the mechanisms/pathways involved in attenuating diabetes mediated neurobehavioral dysfunction. By affecting the Nrf2 signalling pathways, Met was able to lessen the neurogenesis damage and neurocognitive abnormalities caused by sevoflurane in the growing rat brain (Fan et al. 2023). Sem increases SOD and Nrf2 levels, shielding the mice from oxidative stress brought on by EAE (Sadek et al. 2023).

Rotenone-tempted PD stimulates caspase-3 and cytokine production (Li et al. 2017). Met and/or Sem attenuated rotenone-induced alterations by decreasing the cytokine levels including TNF-α, and caspase 3, which point to their antiapoptotic and anti-inflammatory action. Previous studies showed that Met treated diabetic animals has considerably reduced the elevated expression of caspase 3 in the brains as a sign of apoptosis regulation by Met. In addition to its capacity to decrease infarction size and exhibit anti-inflammatory properties, Sem studied to have an anti-apoptotic effect through suppression of the Caspase-3 signalling pathway (Yang et al. 2019).

Met and/or Sem was able to ameliorate DM+PD group histopathological changes within cerebral cortex, fascia dentata and substantia nigra of brain tissues. Mild to moderately reduced and degraded neurons with pyknotic nuclei were seen in the cerebral cortex, fascia dentata, and substantia nigra with only a few dispersed pyknotic neurons with perineural vacuolations and congestion of tiny blood vessels with perivascular edema in neurons of the fascia dentata and substantia nigra which is consistent with previous studies (Sadek et al. 2023; Soliman et al. 2019).

## Conclusion

Individuals diagnosed with diabetes are far more likely to experience mental health issues and compromised brain function. Taken together, the findings from the present study for the first time revealed that the synergistic effects of Met and Sem to establish neuroprotection in PD in male diabetic rats compared to each drug separately. According to the results of this study, the restorative impact of Met and/or Sem against T2DM and PD induced alterations in neurobehavioral and biochemical/molecular indices is attributed to the enhancement of endogenous antioxidant systems, reduced lipid peroxidation, suppression of oxidative/ inflammatory stress, and most importantly regulation of molecular markers of oxidant stress and tissue damage, Nrf2 and caspase 3.

## Data Availability

Data presented in this study are available from the corresponding author upon reasonable request.
